# Malignant transformation of a long-term ulcer in a patient with leprosy sequelae: a case report

**DOI:** 10.1590/1677-5449.202400442

**Published:** 2024-09-03

**Authors:** Aline Gabriele Etur dos Santos, Antônio Augusto Moreira

**Affiliations:** 1 Universidade de Mogi das Cruzes – UMC, Mogi das Cruzes, SP, Brasil.

**Keywords:** leprosy, surveillance, wounds, ulcer, malignant tumor, carcinoma

## Abstract

Marjolin’s ulcer (MU) is a rare condition defined as a malignant skin tumor arising from chronic wounds and inflammation. The most common histological findings in MUs are squamous cell carcinomas (SCC) (or spinocellular carcinomas [SPC]), such as basal cell carcinomas (BCC) and malignant melanomas (MM). The aim of this study is to report the case of a patient with sequelae of leprosy who presented malignant transformation in a long-standing ulcer on the right leg, thereby contributing to understanding of the progression and significance of early diagnosis of MU. The MU was diagnosed by incisional biopsy of the lesion and upon obtaining a positive result the patient was referred to an oncology service. Treatment of MU is multidisciplinary and surgical excision is the first therapeutic option. Proper management and surveillance of chronic ulcers by the healthcare team are necessary for early recognition of MU.

## INTRODUCTION

Malignant transformation of a chronic ulcer, also known as Marjolin’s ulcer (MU), is a rare condition defined as a malignant tumor arising on the skin after chronic wounds and inflammation.^1,2^ The most common histological findings in MUs are squamous cell carcinomas (SCC) (or spinocellular carcinomas [SPC]), generally secondary to burns. However, other types of tumor may also occur, such as basal cell carcinomas (BCC) and malignant melanomas (MM), all of which are differentiated and have a high potential for malignancy.^3^

The objective of this study is to report the case of a patient with Hansen’s disease sequelae who presented with malignant transformation of a long-term ulcer on the right lower limb (RLL), thereby contributing to understanding of MU progression and of the importance of early diagnosis. The information presented in this study was obtained by review of the patient’s medical records, which contained photographs, and from a review of the literature. This is a descriptive study in the case report format. Free and informed consent was obtained in writing from the patient. The study was submitted to the Human Research Ethics Committee at the Universidade de Mogi das Cruzes, under Ethics Appraisal Submission Certificate 68526123.4.0000.5497, and was granted approval under decision number 6.027.899.

## CASE DESCRIPTION

The patient was a 58-year-old white male with Hansen’s disease sequelae. He was a resident of the city of Mogi das Cruzes, in the state of São Paulo, Brazil, and presented at a healthcare center affiliated to a Hansen’s disease control program in a town in the region on January 4, 2023, on referral from his previous physician. He described worsening pain from an ulcer on the posterior surface of the RLL, which had existed for at least 20 years, and which had changed in appearance over the previous 2 months. Physical examination found ulceration of approximately 8 cm in diameter, with a dark granular base, irregular pale borders, a moderate quantity of seropurulent secretion, and mild hyperemia of the surrounding area, which are abnormal findings for the characteristics of a chronic venous ulcer. Antibiotic testing found ciprofloxacin-sensitive *Pseudomonas spp.*, and the patient was prescribed ciprofloxacin 500 mg, at one tablet every 12 hours, orally, for 10 days. The wound was dressed with collagenase and chloramphenicol.

After some days, biopsy specimens were taken from the margin and base of the wound because of suspected malignancy, since the appearance had worsened since the patient’s first physical examination, and the changes found were not compatible with a chronic venous ulcer. On February 8, 2023, the biopsy report detailed atypical findings from the base of the ulcer that could correspond to reactive changes, but did not rule out malignancy. A wedge biopsy was therefore taken from the margin of the lesion, an X-ray of the RLL was ordered, and the patient was prescribed amoxycillin with clavulanate 875 + 125 mg, every 12 hours, for 14 days, and paracetamol 500 mg, to be taken if in pain.

**Figure 1:**
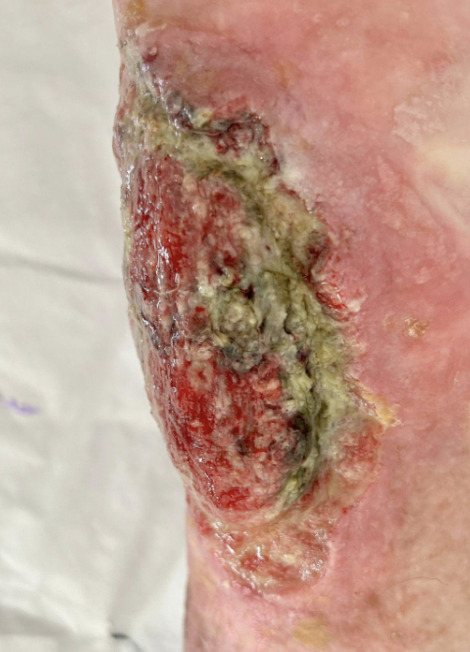
Macroscopic characteristics of the ulcer, showing presence of lesions and irregular and pale margins.

**Figure 2:**
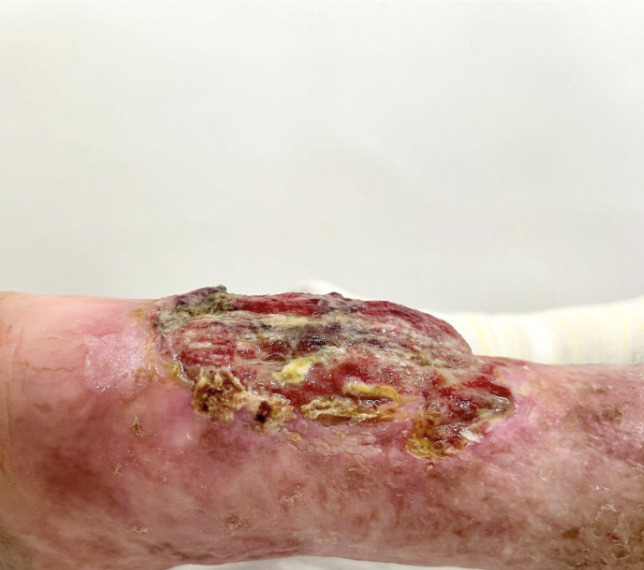
Macroscopic characteristics of the ulcer, showing the raised base of the ulcer.

On February 11, 2023, a pathology report was received containing a diagnosis of invasive grade 1 SPC (histological), extending to the surfaces of the specimen, which measured 1.1 x 0.8 x 0.7 cm and had a whitened and rough epidermal surface (Figures 1 and 2). The X-ray result did not show any destruction of bone in the RLL. After confirmation of the diagnosis, the patient was referred to an oncology service, where inguinal node involvement was detected and inguinal lymphadenectomy was performed, without complications. The patient died from acute respiratory failure before tumor staging was concluded or treatment had been initiated.

## DISCUSSION

Marjolin’s ulcer was first described in 1828 by the French surgeon Jean Nicholas Marjolin, who defined it as a benign cancroid lesion. Years later, Dupuytren observed that this type of ulcer was the result of malignant transformation of chronic wounds.^4^ Nowadays, MU is classified as a rare cutaneous malignancy, primarily triggered in skin with chronic wounds and burn scars.^5^ neoplasm of epithelial origin (defined as SCC and also known as SPC) is identified in around 86% of cases. This type is followed by CBC, seen in approximately 10% of cases and, more rarely, by melanoma, sarcoma, dermatofibrosarcoma, mucoepidermoid carcinoma, and leiomyosarcoma.^6^ According to data from Brazil’s National Cancer Institute (INCA), non-melanoma skin cancer, which includes SCC and CBC, is the most common in Brazil and accounts for around 30% of all malignant tumors registered in the country.^7^

Certain chronic inflammatory diseases have been reported as factors that make development of MU more likely, including traumatic injuries, ulcers caused by chronic venous insufficiency, and osteomyelitis. Additionally, there is a high risk of development in immunocompromised individuals, and around 90% of cases of MU are secondary to burns.^1,2^ Rarer situations can also occur, as shown in a study by Yuste et al., who reported a curious case of development of MU in a laparotomy scar.^8^ Burn scars are the conditions most frequently reported as triggering malignant transformation, which is found in 0.7 to 2% of such scars.^6^

The mechanism involved in malignant transformation of a wound has not yet been fully elucidated and is characterized as multifactorial. It is believed that the cells of scar tissue release many pro-mitotic toxins and the injured area becomes a site that is protected from recognition by the immune system because of reduced vascularization and lymphatic drainage, preventing adequate defense against aggressions.^9,10^ Alternatively, malignant cells may emerge under the influence of chemical or viral carcinogens, or because of spontaneous or hereditary genetic mutations.

Studies report that people who have the Li-Fraumeni Syndrome, i.e. carriers of a mutation on the TP53 gene, may develop SPC more frequently and that mutations on the FAS gene may be related to development of MU on burn scars.^10,11^

Thio et al.^4^ describe certain characteristics of wounds that make it possible to diagnose MU more easily. These are: sudden appearance of scabs or ulceration on the scar, sudden increase in local pain and of the size of the scar/ulcer, presence of bleeding, unexpected delay in healing of small injuries, increased exudate and discharge, and failure to heal for more than 2 years.^4^ Additionally, many invasive and higher histological grade MUs may also exhibit bone involvement, such as pathological fractures and/or destruction. It is therefore important to assess the internal status of the MU, with X-rays of the area, as were requested for the patient in this report.^9^

Treatment of MU is multidisciplinary and the first-choice treatment option is surgical intervention when possible. Generically, this includes Mohs surgery, which consists of ample excision of the site with well-defined margins and, in some cases, may extend to amputation of the involved limb. Additionally, lymph node dissection may be considered in the presence of palpable lymph nodes, depending on the findings of an ultrasound examination and sentinel lymph node biopsy, especially when malignant melanoma has been diagnosed.^6^ Chemotherapy and radiotherapy are recommended in cases with metastases to regional lymph nodes, high grade lesions (III), lesions larger than 10 cm in diameter, and when surgery is not possible.^9^

The time to malignant transformation of a wound varies greatly between different studies, ranging from 1 month to 64 years, with an approximate mean of 36 years. When the time to transition is less than 1 year, it is classified as acute MU, with a predominance of CBC, while cases that have onset after more than 1 year are defined as chronic MU and are generally associated with SPC.^9^ Progression is primarily dependent on the type of trauma that occurs to cause the scar and the patient’s health and immune system conditions, such as the presence of certain diseases that can shorten the time interval.^12^

Hansen’s disease, also known as leprosy, is a chronic disease caused by *Mycobacterium leprae*, that can damage the peripheral nervous system and, secondarily, the skin and other tissues, and is very often associated with neuropathic ulcers.^13^ The presence of chronic wounds in patients, particularly those with diseases associated with peripheral neuropathies such as Hansen’s disease, requires adequate and routine supervision and follow-up. It is essential that they receive care from a multidisciplinary health care team, including vascular physicians, oncologists and dermatologists, nurses, and nursing technicians and auxiliaries, to ensure that if there is any sudden change in the characteristics of the wound, a biopsy is performed, so that it can be diagnosed and treated as early as possible.
