# Supplementary material for: Malignant transformation of a long-term ulcer in a patient with leprosy sequelae: a case report
Source: J Vasc Bras. 2024 Sep 3;23:e20240044. doi: 10.1590/1677-5449.202400442 (PMC11404777; doi:10.1590/1677-5449.202400442)
Supplement: Translation

# Translation: Malignização de úlcera de longa duração em paciente com sequela de hanseníase: um relato de caso

**DOI:** 10.1590/1677-5449.202400441

**Authors:** Aline Gabriele Etur dos Santos, Antônio Augusto Moreira

**Affiliations:** 1 Universidade de Mogi das Cruzes – UMC, Mogi das Cruzes, SP, Brasil.

**Keywords:** hanseníase, vigilância, feridas, úlcera, tumor maligno, carcinoma

## Abstract

A úlcera de Marjolin (UM) é uma condição rara definida como um tumor maligno na pele decorrente de feridas crônicas e inflamação. Os achados histológicos mais comuns nas UMs são os carcinomas de células escamosas (CCE) ou carcinomas espinocelulares (CEC), como carcinomas basocelulares (CBC) e melanomas malignos (MM). O objetivo deste estudo é relatar o caso de um paciente com sequela de hanseníase que apresentou malignização de sua úlcera em perna direita de longa duração e, a partir disso, compreender a progressão e a importância do diagnóstico precoce da UM. O diagnóstico da UM foi realizado mediante biópsia incisional da lesão e, após obtido o resultado positivo, o paciente foi encaminhado a um serviço de Oncologia. O tratamento da UM é multidisciplinar, sendo a abordagem cirúrgica a primeira opção terapêutica. O manejo adequado e a vigilância de úlceras crônicas pela equipe de saúde tornam-se necessários para o reconhecimento precoce da UM.

## INTRODUÇÃO

A malignização de uma úlcera crônica, também conhecida como úlcera de Marjolin (UM), é uma condição rara definida como um tumor maligno na pele associado a inflamação e feridas crônicas^1,2^ . Os achados histológicos mais comuns nas UMs são os carcinomas de células escamosas (CCE) ou carcinomas espinocelulares (CEC), geralmente decorrentes de queimaduras. Porém, podem ocorrer também outros tumores, como carcinomas basocelulares (CBC) e melanomas malignos (MM), sendo todos diferenciados e com um alto potencial de malignidade^3^ .

Este estudo tem como objetivo relatar o caso de um paciente com sequela de hanseníase que apresentou malignização de sua úlcera em membro inferior direito (MID) de longa duração e, com isso, compreender a progressão e a importância do diagnóstico precoce da UM. As informações contidas neste trabalho foram obtidas por meio de revisão do prontuário do paciente, o qual continha registros fotográficos e revisão de literatura. Trata-se de um estudo descritivo do tipo relato de caso. Foi obtida a assinatura do Termo de Consentimento Livre e Esclarecido pelo próprio paciente. O estudo foi submetido ao Comitê de Ética em Pesquisa com Seres Humanos da Universidade de Mogi das Cruzes com Certificado de Apresentação de Apreciação Ética 68526123.4.0000.5497, obtendo parecer de aprovação de número 6.027.899.

## DESCRIÇÃO DE CASO

O paciente era do sexo masculino, 58 anos de idade, branco, e apresentava sequela de hanseníase. Morador da cidade de Mogi das Cruzes, no estado de São Paulo, compareceu a uma Unidade de Atenção aos Programas de Saúde em uma cidade localizada no Alto Tietê no dia 4 de janeiro de 2023, após ter sido encaminhado pelo médico anterior. Relatava piora da dor na úlcera em face posterior do MID, a qual já possuía há mais de 20 anos, e mudança de seu aspecto nos últimos dois meses. Ao exame, foi constatada uma ulceração de aproximadamente 8 cm de diâmetro, com fundo granuloso escuro, irregularidade e clareamento de bordos, saída de moderada quantidade de secreção seropurulenta e hiperemia branda ao redor, características que evidenciam achados anormais encontrados em úlcera venosa crônica. O antibiograma realizado apresentou *Pseudomonas spp.* sensível a ciprofloxacino, sendo o paciente medicado com ciprofloxacino 500 mg, com um comprimido de 12 em 12 horas, por via oral, por 10 dias. Além disso, foi realizado curativo com colagenase associado a cloranfenicol.

Após alguns dias, foi realizada uma biópsia do bordo e do fundo da lesão por suspeita de malignidade, visto que houve uma piora dos aspectos evidenciados no primeiro exame físico realizado com o paciente, que não eram compatíveis com os de uma úlcera venosa crônica habitual. Em 8 de fevereiro de 2023, o laudo da biópsia apresentou atipias que poderiam corresponder às alterações reativas de fundo da úlcera, porém não foi excluída a possibilidade de malignidades. Diante disso, foi realizada uma nova biópsia da borda da lesão em cunha, solicitada uma radiografia do MID e prescrito amoxicilina com clavulanato 875 + 125 mg, de 12 em 12 horas, por 14 dias, e paracetamol 500 mg, em caso de dor.

No dia 11 de fevereiro de 2023, foi obtido o relatório anatomopatológico com diagnóstico de CEC grau 1 (histológico) invasivo com extensão às faces cruentas do espécime, o qual media 1,1 x 0,8 x 0,7cm, com superfície epidérmica esbranquiçada e rugosa (Figuras 1 e 2). O resultado da radiografia não apresentou destruição óssea no MID. Após a confirmação do diagnóstico, o paciente foi encaminhado a um serviço de Oncologia, onde foi apresentado envolvimento ganglionar inguinal e realizada uma linfadenectomia inguinal sem complicações. O paciente veio a falecer por insuficiência respiratória aguda antes da conclusão do estadiamento do tumor e do início do tratamento.

**Figura 1:** Característica macroscópica da úlcera, evidenciando a presença de lesões, irregularidade e clareamento de bordos.

**Figura 2:** Característica macroscópica da úlcera, evidenciando o fundo da úlcera elevado.

## DISCUSSÃO

A UM foi descrita pela primeira vez pelo médico cirurgião francês Jean Nicholas Marjolin no ano de 1828, o qual a denominou de uma cancroide sem natureza maligna. Anos depois, Dupuytren observou que esse tipo de úlcera era uma malignização de feridas crônicas^4^ . A UM, atualmente, é caracterizada como uma malignidade cutânea rara desencadeada em peles que apresentam, principalmente, feridas crônicas e cicatrizes de queimaduras^5^ . Em cerca de 86% dos casos, identifica-se a neoplasia de origem epitelial denominada CCE, também conhecida como CEC. Este é seguido pelo CBC em aproximadamente 10% dos casos e, mais raramente, por melanoma, sarcoma, dermatofibrossarcoma, carcinoma mucoepidermóide e leiomiossarcomas^6^ . Segundo dados do Instituto Nacional de Câncer (INCA), o câncer de pele não melanoma, o qual inclui o CCE e CBC, é o mais frequente no Brasil e corresponde a cerca de 30% de todos os tumores malignos registrados no país^7^ .

Algumas doenças inflamatórias crônicas foram relatadas como fatores que propiciam o desenvolvimento da UM, dentre elas feridas traumáticas, úlceras por insuficiência venosa crônica e osteomielite. Além disso, há um alto risco de desenvolvimento em indivíduos imunocomprometidos, sendo que cerca de 90% dos casos de UM são decorrentes de queimaduras^1,2^ . Situações mais raras podem ocorrer, como mostra um estudo de Yuste et al., que relatou um curioso caso do desenvolvimento da UM em uma cicatriz de laparotomia^8^ . As cicatrizes por queimaduras são as condições mais frequentemente relatadas como desencadeadoras de malignização, sendo encontrada em 0,7 a 2% dessas cicatrizes^6^ .

O mecanismo envolvido na malignização de uma ferida ainda não está completamente elucidado, sendo caracterizado como multifatorial. Acredita-se que as células do tecido cicatricial liberam muitas toxinas pró-mitóticas e que a área da lesão se torna um sítio protegido do reconhecimento pelo sistema imunológico devido à reduzida vascularização e drenagem linfática, impossibilitando a defesa adequada contra agressões^9,10^ . Outrossim, as células malignas podem surgir sob a influência de carcinógenos químicos ou virais e por mutações genéticas espontâneas ou hereditárias.

Estudos relatam que portadores da Síndrome de Li-Fraumeni, mutação no gene TP53, podem desenvolver CEC com maior frequência, e que mutações no gene FAS podem estar relacionadas com o desenvolvimento de UM em feridas de queimaduras^10,11^ .

O estudo de Thio et al.^4^ relatou algumas características de uma ferida que possibilitam diagnosticar com maior facilidade a UM. São elas: o aparecimento repentino de crostas ou ulceração na cicatriz, o aumento repentino da dor local e do tamanho da cicatriz/úlcera, presença de sangramentos, atraso inesperado na cicatrização de pequenas feridas, aumento do exsudato e de corrimento e falha na cicatrização por mais de 2 anos^4^ . Ademais, muitas UM podem apresentar acometimento ósseo, como a destruição e/ou fraturas patológicas, em graus histológicos maiores e invasivos. Por isso, torna-se importante uma avaliação da condição da UM internamente, por meio de exames de radiografia da região, como foi pedido para o paciente deste relato^9^ .

O tratamento da UM é multidisciplinar, sendo a abordagem cirúrgica a primeira opção terapêutica, quando possível. Genericamente, inclui a cirurgia de Mohs, a qual consiste em uma excisão ampla do local com margens bem definidas e que, em alguns casos, pode levar à amputação do membro acometido. Ademais, na presença de linfonodos palpáveis, pode ser considerada a dissecção linfonodal, dependendo dos achados do exame ultrassonográfico e da biópsia do linfonodo sentinela, especialmente quando se tem o diagnóstico de melanoma maligno^6^ . A quimioterapia e radioterapia são recomendadas em situações em que há metástases de linfonodos regionais, lesões de alto grau (III), lesões maiores de 10 cm de diâmetro e quando não é possível a realização da cirurgia^9^ .

O tempo para que ocorra a malignização de uma ferida é extremamente variável entre os estudos, variando de 1 mês a 64 anos, com uma média de, aproximadamente, 36 anos. Quando o tempo de transição é inferior a 1 ano, é denominada como UM aguda, com predominância de CBC, enquanto acima de 1 ano é definida como UM crônica, e geralmente associada ao CEC^9^ . Essa progressão depende, principalmente, do tipo de trauma ocorrido sobre a cicatriz e da condição de saúde e do sistema imunológico do paciente, como a presença de determinadas doenças, que podem encurtar esse intervalo de tempo^12^ .

A hanseníase, também conhecida como lepra, é uma doença crônica ocasionada pela *Mycobacterium leprae*, capaz de danificar o sistema nervoso periférico e, secundariamente, a pele e outros tecidos, sendo muito comumente associada à presença de úlceras neuropáticas^13^ . A presença de feridas crônicas em pacientes, principalmente com doenças associadas às neuropatias periféricas como a hanseníase, necessita de uma supervisão e acompanhamento adequados e rotineiros. Torna-se essencial o cuidado e a abordagem da equipe de saúde multiprofissional, incluindo médicos vasculares, oncologistas e dermatologistas, enfermeiros e técnicos e auxiliares de enfermagem, para que, caso haja alguma alteração repentina da característica da ferida, seja realizada a biópsia da lesão, para que esta seja diagnosticada e tratada o mais precocemente possível.
